# Adverse Event Profiles of the Third-Generation Aromatase Inhibitors: Analysis of Spontaneous Reports Submitted to FAERS

**DOI:** 10.3390/biomedicines12081708

**Published:** 2024-08-01

**Authors:** Yina Zhang, Lingzhu Zhao, Yanning Liu, Jingkang Zhang, Luyan Zheng, Min Zheng

**Affiliations:** State Key Laboratory for Diagnosis and Treatment of Infectious Diseases, National Clinical Research Center for Infectious Diseases, Collaborative Innovation Center for Diagnosis and Treatment of Infectious Diseases, The First Affiliated Hospital, College of Medicine, Zhejiang University, 79 Qingchun Road, Hangzhou 310003, China

**Keywords:** aromatase inhibitors, breast cancer, FAERS, adverse event

## Abstract

The third-generation aromatase inhibitors (AIs), represented by letrozole, anastrozole, and exemestane, have been used as a standard first-line adjuvant therapy for postmenopausal breast cancer patients with positive hormone receptor. However, their safety in the real world has not been systematically analyzed. We used the U.S. Food and Drug Administration Adverse Event Reporting System (FAERS) to investigate adverse event (AE) profiles of the three AIs, covering the period from Q1 2004 to Q3 2023. The time-to-event onset profiles and cumulative incidence were analyzed by Weibull shape parameter test and Kaplan–Meier method, respectively. The disproportionality analysis was utilized to assess drug toxicity risk. Based on the FAERS database, 18,035, 8242, and 7011 reports listing letrozole, anastrozole, and exemestane as primary suspected drugs were extracted, respectively. AEs associated with anastrozole displayed the latest onset (*p* < 0.0001); meanwhile, WSP test showed that all three AIs had early failure-type profiles. At the preferred term level, we acquired 95, 59, and 42 significant signals associated with letrozole, anastrozole, and exemestane, which involved 18, 13, and 15 system organ classes, respectively. The three AIs all reported that their strongest AE signal was trigger finger. Neutropenia was the most frequent AE for letrozole, while the highest occurrences of anastrozole and exemestane were arthralgia. We also found that interstitial lung disease, a rare but serious AE, showed strong signal intensity in all three AIs. Additionally, letrozole was also associated with lots of other rare but serious AEs in hematologic, respiratory, and hepatic systems, which were not recorded in the instructions. Our analysis of safety warning signals of the third-generation AIs from the FAERS database provided reference for clinical safe and rational drug use.

## 1. Introduction

Aromatase, also called estrogen synthetase, is a complex enzyme belonging to the cytochrome P450 family [1]. It is abundantly expressed in gonadal and extra-gonadal tissues, including ovary, testis, placenta, brain, adipocytes, and so on [2]. Aromatase is also the key and rate-limiting enzyme as a catalytic role in estrogen biosynthesis by converting androgens into estrogens [3]. Disruption in the homeostasis of aromatase, along with either decreased or increased estrogen levels, can lead to a diverse range of illnesses and adverse reactions [4]. Excessive estrogen production is positively correlated with breast cancer (BC), which is one of the prevalent malignancies in women globally [5]. Estrogen in premenopausal females primarily originates from their ovaries, whereas in postmenopausal females, estrogen is predominantly synthesized by aromatase generated by extra-gonadal tissues [6]. For the latter with BC, aromatase inhibitors (AIs) are crucial drugs which dramatically reduce estrogenic levels in the blood by specifically causing the inactivation of aromatase, blocking the aromatization, and inhibiting estrogen formation, so as to achieve the purpose of curing the BC [6].

As a novel therapy for BC, AIs have attracted the attention of many academics. So far, the market has witnessed the development and the launch of three generations of AIs, categorized as steroid and non-steroid inhibitors based on their structures [5]. Steroidal AIs include exemestane and formestane, and non-steroidal AIs include aminoglutethimide, letrozole, and anastrozole [5]. Initially developed as an antiepileptic medication, aminoglutethimide, known as the first-generation AI, was subsequently discovered to suppress aromatase activity, resulting in decreased estrogen levels [5]. Formestane, also known as 4-hydroxy-androstenedione (4-OH-A), was recognized as the second-generation AI and served as the first selective AI against BC [3,5]. Currently, the first-line endocrine treatment of choice for BC in postmenopausal women mainly includes letrozole, anastrozole, and exemestane, which are nominated as the third-generation AIs [5]. Compared with aminoglutethimide and formestane, the third-generation AIs have outstanding advantages in clinical practice because they are highly selective for aromatase, well tolerated, and eutherapeutic [7,8].

The widespread application of the third-generation AIs has led to increased public concern regarding their drug-related adverse events (AEs), among which hot flushes, muscle and joint pain, osteoporosis, dyslipidemia, cardiovascular events, fatigue, dizziness, headache, nausea, sweating, vaginal dryness, and so on are common AEs mentioned in the drug instruction. However, some serious AEs may go unnoticed because of the restrictions imposed by premarketing clinical trials, including rigorous trial protocols, exclusion criteria, and limited duration of follow-up. The U.S. Food and Drug Administration (FDA) Adverse Event Reporting System (FAERS) database, established by the FDA, is a publicly accessible system where individuals can report AEs related to FDA-approved drugs. Its purpose is to monitor the safety of these drugs after post-marketing and to identify any potential connections between the drugs and AEs in the real world [9]. Our study employs data mining techniques to identify and examine the AEs linked to the third-generation AIs based on the FAERS database. It aims to raise awareness and encourage further investigation into this issue, while also offering insights for clinical drug management and rational drug utilization.

## 2. Methods

### 2.1. Data Sources and Mining

Our data was downloaded from the FAERS database, which is a useful tool for the FDA to identify new safety issues that may be associated with a marketed drug. A wide range of AE data is available in the FAERS database, which covers the period from January 2004 to the present and is regularly updated for each quarter. Users can search AE data from the FAERS Public Dashboard, a highly interactive web-based tool, or directly obtain raw data by downloading the FAERS Quarterly Data Extraction Files on the official website. The informatic structure of the FAERS database complies with the international safety reporting guidance issued by the International Conference on Harmonization (ICH E2B). Overall, the dataset of FAERS database contains seven data files: (1) patient demographic and administrative information (DEMO file), (2) drug/biologic information (DRUG file), (3) adverse events (REAC file), (4) patient outcomes (OUTC file), (5) report sources (RPSR file), (6) drug therapy start and end dates (THER file), and (7) indications for use/diagnosis (INDI file) [10].

Specifically, we extracted all AEs related to the three AIs from the FAERS database, covering the period from the first quarter of 2004 to the third quarter of 2023. The deduplication process recommended by the FDA was followed to eliminate duplicate records. In the DEMO file, we selected PRIMARYID, CASEID, and FDA_DT fields, and arranged them in the sequence of CASEID, FDA_DT, and PRIMARYID. We kept the report with the highest FDA_DT value for cases with the same CASEID, and retained the report with the highest PRIMARYID value for cases that have the same CASEID and FDA_DT. The code assigned to each drug for its reported role in the event in the DRUG file can be divided into four categories: PS (primary suspect drug), SS (secondary suspect drug), C (concomitant), and I (interacting). For this research, we exclusively analyzed monotherapy and chose the reports with the role_cod that was specifically identified as PS in the DRUG file. The data was retrieved using the keywords “Letrozole/Femara/CGS 20267” and “Anastrozole/Arimidex/ZD 1033/ICI D1033” and “Examestane/Aromasil/Aromasin/Aromasine/FCE 24304” for collecting patient demographics, drug information, AE information, patient outcomes, and so on to further statistically analyze. All valid AIs-related AEs in our study were coded using preferred terms (PTs) and grouped into the System Organ Class (SOC) according to the Medical Dictionary for Regulatory Activities (MedDRA) version 26.1.

### 2.2. Statistical Analysis

The disproportionality analysis is a widely used analytical tool in pharmacovigilance [11,12]. In our study, to acquire more dependable results, we implemented two algorithms to measure disproportionality: the reporting odds ratio (ROR) and proportional reporting ratio (PRR). The ROR is the ratio of between the odds of reporting a targeted AE exposed to a targeted drug divided by the corresponding odds for all other drugs in the database [13]. The PRR is the proportion of spontaneous reports for a targeted drug that are linked to a specific AE, divided by the corresponding proportion for all other drugs in the database [14]. Tables S1 and S2 provided a summary of the primary algorithms utilized for calculating the signal strength value that indicated the correlation between the interested drug and a particular AE of interest. In the equation, a represents the number of targeted AEs of a given drug, b represents the number of other AEs of a given drug, c represents the number of targeted AEs of other drugs, and d represents the number of other AEs of other drugs. Based on the ROR and PRR formulas to perform the calculations, when the criteria outlined in Table S2 was satisfied at the same time, an AE would be deemed closely linked to the administration of the interested drug, with greater values indicating a more robust statistical correlation. Data regarding gender, age, weight, reporter, geographical distribution, and outcome of AEs was displayed using descriptive statistics, and the count data was expressed as frequency (%). Furthermore, the median duration, interquartile range (IQR), and Weibull shape parameter (WSP) test were used to analyze the time-to-event onset data, which referred to the duration between treatment initiation and the occurrence of AEs. For comparison of multiple groups in the difference of time-to-onset data, the nonparametric Kruskal–Wallis test was used and *p* < 0.05 was considered statistically significant. WSP test has previously been proposed as a method for determining the varying ratio of incidence of AEs, which depends on the drug mechanism of action and often varies over time [15,16,17]. In addition, the hazard functions of AEs can also be evaluated by fitting a Weibull model to time-to-event onset data [18]. Based on two observation indexes, shape parameter β and 95% confidence interval (CI), WSP test divides the variation of hazard occurrence over time into three types. The first type is early failure-type profile (β < 1, 95% CI < 1), which indicates that the hazard increases in the early stage of treatment and then gradually decreases over time. The second type is random failure-type profile (β was equal to or nearly 1, 95% CI included the value 1), which refers to the constant occurrence of hazard over time. The third type is wear-out failure-type profile (β > 1, 95% CI excluded the value 1), which refers that the hazard occurs at an increased rate [15,16,17]. Then Kaplan–Meier method was utilized to draw cumulative incidence curves of AEs associated with letrozole, anastrozole, and exemestane, and the difference was analyzed using a comparison method based on a log-rank test (*p* < 0.05 was considered statistically significant). Subgroup analysis results were confirmed using signals of disproportionality reporting methods and the forest plot. All analyses were performed using R software version 4.3.1 (The R Foundation for Statistical Computing, Vienna, Austria).

### 2.3. Signal Screening

In this study, in order to obtain higher-frequency AEs and reduce the occurrence of false positive signals, we screened all three AIs-related events with reporting volume ≥ 30 case reports in the database. AEs obviously unrelated to the interested drug or affected by the primary disease were considered to be excluded. The former were mainly associated with several System Organ Class (SOC) categories, involving soc_name_cn as injury, poisoning and procedural complications; product issues; social circumstances; pregnancy, puerperium, and perinatal conditions; surgical and medical procedures; and congenital, familial, and genetic disorders in the REAC file.

## 3. Results

### 3.1. Population Characteristics

As of the third quarter of 2023, a total of 33,288 reports of AIs were submitted to FAERS, including 18,035 reports for letrozole, 8242 reports for anastrozole, and 7011 reports for exemestane, corresponding to 72,554, 29,667, and 21,945 reported AEs, respectively. The clinical characteristics of patients treated with AIs were described in Table 1. Through data mining, we found that the number of AEs was much larger than the number of patients, suggesting that more than one AE might occur in a patient with the AI treatment. The majority of patients in the reports were female, with letrozole accounting for 91.06%, anastrozole for 95.98%, and exemestane for 92.24%. In most of these reported cases, the age and weight data of patients were missing. According to the available data, the median age of reported cases was 64 years (IQR 54–73) for letrozole, 66 years (IQR 59–74) for anastrozole, and 67 years (IQR 59–74) for exemestane, respectively. Similarly, the median weight was 68 kg (IQR 59–80) for letrozole, 69.90 kg (IQR 60–81.60) for anastrozole, and 70 kg (IQR 60.72–82.18) for exemestane, respectively. Based on whether the occupation of a reporter was significantly associated with medicine specialty, we divided all reporters who submitted AEs to the FAERS database into three subgroups: medical professional, non-medical professional, and unknown. The medical professional subgroup consisted of physicians, pharmacists, and other health professionals, while the non-medical professional subgroup consisted of consumers and lawyers. The others reporting missing data of occupation were incorporated into the unknown subgroup. The most frequent reporter of letrozole and exemestane AEs was medical professional (64.96% and 51.21%, respectively), but that of anastrozole was non-medical professional. The records were reported from more than 100 countries and were primarily submitted by the USA, 25.60% for letrozole, 72.76% for anastrozole, and 57.47% for exemestane, while the reports submitted by patients who received letrozole were relatively scattered. In addition, the data also showed that hospitalization was the main reported outcome for the three AIs-related AEs (letrozole, n = 4519; anastrozole, n = 1294; exemestane, n = 1389).

### 3.2. Time-to-Event Onset Analysis and Cumulative Incidence

Table 2 summarized the time-to-event onset data of the three AIs-associated AE reports and the corresponding WSP test results, of which 5228, 1999, and 1533 patients reported on occurrence time of letrozole, anastrozole, and exemestane-associated events, respectively. The median (IQR) time-to-event onset of the three AIs-associated AEs was 99.50 (IQR 25.00–431.00), 268.00 (IQR 61.00–791.00), and 116.00 (IQR 31.00–437.00) days, respectively. From the data, it was observed that the AEs mainly occurred after a longer period after the first dose of anastrozole (Kruskal–Wallis test, *p* < 0.0001). Meanwhile, the occurrence time of AEs differed between letrozole-treated and exemestane-treated patients (Kruskal–Wallis test, *p* = 0.019). In addition, we conducted a WSP test for three AIs-associated AE reports, and we found that letrozole, anastrozole, and exemestane-associated AEs all suggested early failure-type profiles.

**Figure 1:**
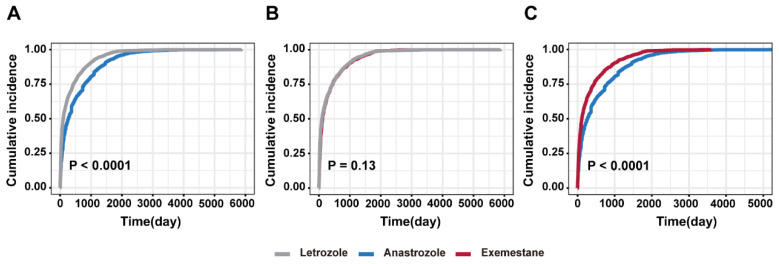
Comparison of cumulative incidences of AEs. (**A**) Letrozole vs. Anastrozole, *p* < 0.0001. (**B**) Letrozole vs. Exemestane, *p* = 0.13. (**C**) Anastrozole vs. Exemestane, *p* < 0.0001. Abbreviations: AE: adverse event.

Kaplan–Meier curves for AEs associated with letrozole, anastrozole, and exemestane were on display in Figure 1. We found a distinct difference of cumulative incidence of AEs between patients receiving anastrozole and those receiving letrozole and exemestane (log-rank test, *p* < 0.0001), whereas the cumulative incidence of AEs presented no obvious difference between letrozole and exemestane (log-rank test, *p* = 0.13).

### 3.3. Disproportionality Analyses for Letrozole, Anastrozole, and Exemestane

Excluding the AEs obviously unrelated to the drug, affected by the primary disease as well as reporting frequency <30, the top 30 AEs with the strongest signal intensity and the highest frequency for the three AIs were analyzed in Tables S3–S8. Letrozole involved 95 positive signals, of which trigger finger had the strongest signal intensity (n = 112, ROR = 25.01), followed by skin hypopigmentation (n = 51, ROR = 24.88) and bone lesion (n = 76, ROR = 18.06). Neutropenia (n = 1843, ROR = 12.49) and arthralgia (n = 1341, ROR = 2.84) reported the top 2 highest AE cases for letrozole. Likewise, anastrozole involved 59 positive signals, and the top 3 AEs with trigger finger (n = 131, ROR = 72.25), vulvovaginal dryness (n = 87, ROR = 57.31) and hot flush (n = 710, ROR = 21.71) showed the strongest signal intensity. Exemestane involved 42 significant signals, of which trigger finger was the strongest signal (n = 37, ROR = 26.71), followed by hot flush (n = 261, ROR = 10.58) and bone pain (n = 190, ROR = 9.03). Surprisingly, the three AIs all reported that their AE with the strongest signal intensity was trigger finger, whereas this AE term had more than twice the ROR in patients treated with anastrozole. Arthralgia was the most commonly reported AE for anastrozole (n = 1215, ROR = 6.45) and exemestane (n = 606, ROR = 4.29). In addition, letrozole involved many rare but serious AEs that were reported by limited literatures, such as neutropenia, leukopenia (n = 635, ROR = 11.11), polyneuropathy (n = 140, ROR = 10.69), hypersensitivity vasculitis (n = 33, ROR = 9.21), thrombocytopenia (n = 527, ROR = 4.09), pulmonary embolism (PE) (n = 272, ROR = 2.30), interstitial lung disease (ILD) (n = 159, ROR = 2.91), and osteonecrosis of jaw (n = 138, ROR = 4.37). Likewise, ILD (n = 72, ROR = 4.36), leukopenia (n = 67, ROR = 3.80), hepatic failure (n = 31, ROR = 2.78), and PE (n = 82, ROR = 2.29) were rare but serious AEs associated with exemestane therapy. Although not mentioned in Table S4, ILD also showed strong signal intensity for anastrozole (n = 47, ROR = 2.10). These rare but severe AEs are newly discovered AE signals, which were not recorded in the instructions.

**Figure 2:**
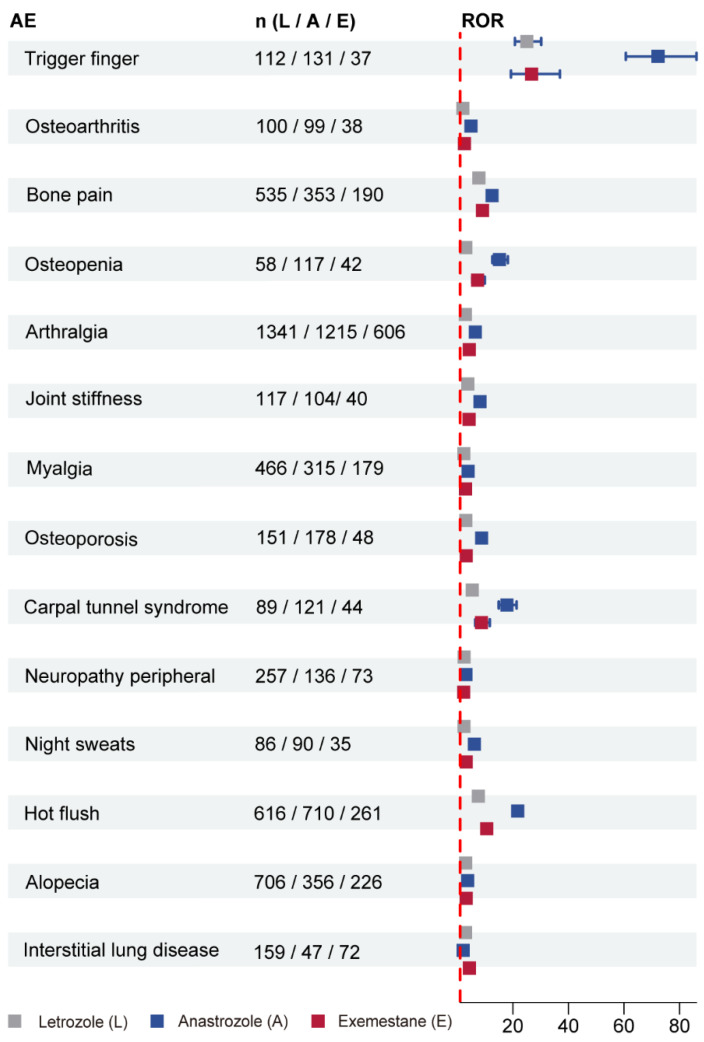
Forest plot of ROR (95% CI) for the same categories of AEs with high frequency and strong signal intensity in the three AIs. Abbreviations: AE: adverse event; ROR: reporting odds ratio; CI: confidence interval; AI: aromatase inhibitor.

Our next study was to assess the differences in common AEs among the three AIs. As shown in Figure 2, these common AEs included trigger finger, osteoarthritis, bone pain, osteopenia, arthralgia, joint stiffness, myalgia, osteoporosis, carpal tunnel syndrome, neuropathy peripheral, night sweats, hot flush, alopecia, and interstitial lung disease, most of which were associated with musculoskeletal toxicity. Except for ILD, each AE term with a lowest reporting frequency was in exemestane. In addition, we found that anastrozole had the highest ROR value in each AE term except for ILD, and letrozole had the weakest association with these common AEs except for ILD and neuropathy peripheral.

### 3.4. SOC Analysis of AEs-Positive Signal Involvement for Three AIs

As indicated in Figure S1 and Tables S9–S11, the SOC results of the three AIs-associated AEs that conformed with the signal screening criteria were analyzed. Although most positive signals associated with letrozole, anastrozole and exemestane belonged to the same SOC category, there were certain differences in some AEs between these three AIs. A total of 18 SOCs were included in the letrozole study, of which sorted by number of signals, the top 5 SOCs included investigations, musculoskeletal and connective tissue disorders, skin and subcutaneous tissue disorders, hepatobiliary disorders, and blood and lymphatic system disorders. Anastrozole involved a total of 13 SOCs, among which a higher percentage of AE positive signals were observed in the categories of musculoskeletal and connective tissue disorders, nervous system disorders, skin and subcutaneous tissue disorders, general disorders, and administration-site conditions, as well as investigations. For exemestane, it had 42 positive signals, encompassing 15 SOCs, of which were highly associated with the system of musculoskeletal and connective tissue disorders, investigations, nervous system disorders, respiratory, thoracic, and mediastinal disorders, general disorders, and administration-site conditions. Additionally, the three AIs had 11 overlapping SOCs, among which AE positive signals displayed a notable majority within the system of musculoskeletal and connective tissue disorders. 

### 3.5. Subgroup Analyses Based on Medical and Non-Medical Professionals

**Figure 3:**
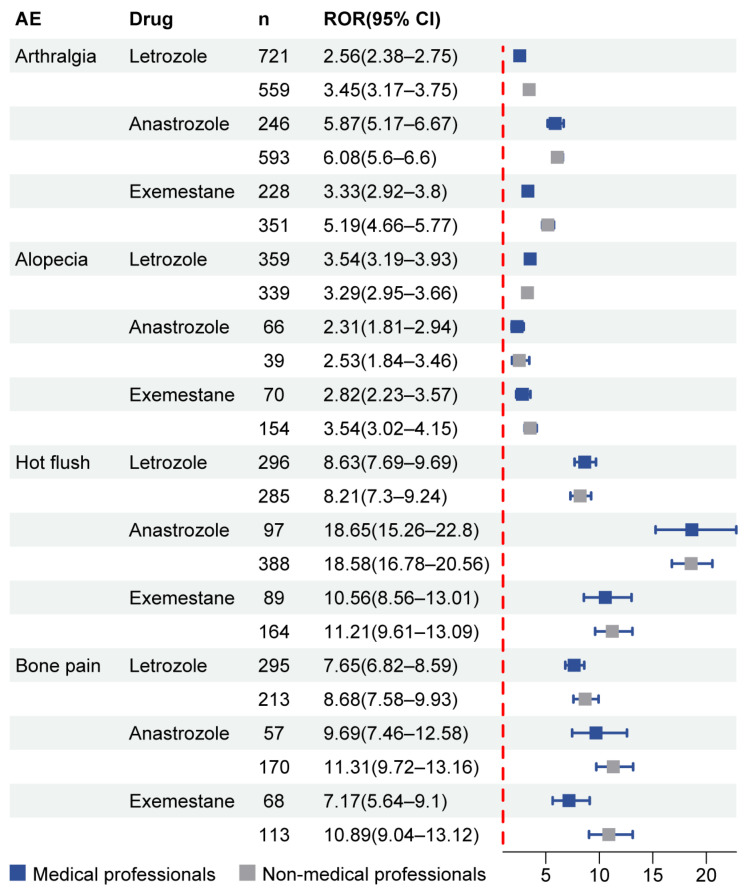
Subgroup analysis of the three AIs showing the forest plot of ROR (95% CI) for the same categories of AEs with high frequency and strong signal intensity. Abbreviations: AE: adverse event; ROR: reporting odds ratio; CI: confidence interval; AI: aromatase inhibitor.

We next aimed to evaluate potential differences in reporting AEs based on subgroup analysis separately for letrozole, anastrozole and exemestane. The AE numbers of letrozole, anastrozole, and exemestane submitted by medical professionals were 46,585, 7051 and 11,355 cases, respectively, but that submitted by non-medical professionals were 23,100, 14,166 and 9753, respectively. Excluding the AE obviously unrelated to the drug, affected by the primary disease as well as reporting frequency <30, letrozole, anastrozole, and exemestane involved 71, 16 and 23 significant signals in the medical professional subgroup, while having 39, 40 and 18 positive signals in the non-medical professional subgroup. Figures S2–S7 showed the disproportionality results for the top 10 AEs with the strongest signal intensity and the highest frequency, respectively. For the medical professional subgroup of letrozole, skin hypopigmentation and neutropenia had the strongest signal intensity and the highest frequency, respectively, while neutropenia and fatigue showed the strongest signal intensity and the highest frequency separately for the non-medical professional subgroup with letrozole use. Likewise, for anastrozole, carpal tunnel syndrome and trigger finger reported the strongest signal intensity separately for the medical and non-medical professional subgroups. Moreover, two subgroups both reported that the most common one was arthralgia with similar ROR values; nonetheless, the number was about twice as high in the medical professional subgroup as in the non-medical professional subgroup. Likewise, for exemestane, the AEs reporting the strongest signal intensity and the highest frequency were the same in both subgroups, hot flashes and arthralgia, respectively. Based on the results of subgroup analysis, we screened out four AEs common to the three AIs, including arthralgia, alopecia, hot flush and bone pain, as shown in Figure 3. The results showed that the ROR values for the four AEs reported by the medical and non-medical professional subgroups were similar in any kind of AI. However, the ROR value of hot flush in the medical and non-medical professional subgroup of anastrozole was approximately twice that of the subgroups of letrozole and exemestane.

## 4. Discussion

In our study, the common AEs of the three AIs all had high frequency and strong signal intensity, among which hot flushes, night sweats, vulvovaginal dryness, sleep disorder, and so on were less harmful to human health, while dyslipidemia and cardiovascular disease (CVD) caused more serious health hazards to the human body. A number of studies revealed that patients receiving AIs were associated with great incidence of developing CVD and increasing blood cholesterol [19,20]. Yet, the cause of the heightened risk of CVD related with the use of AIs was still unclear. On the other hand, the third-generation AIs were confirmed to be correlated with a higher incidence of joint and musculoskeletal toxicity, termed AI–associated musculoskeletal syndrome [21]. Consistently, the same results have been observed in our experiments, in particular, anastrozole accounted for more AE positive signals in the system of musculoskeletal and connective tissue disorders at SOC levels, with the strongest association being with common musculoskeletal AEs compared to the other two drugs. Furthermore, it was discovered that all three AIs consistently reported trigger finger as the AE with the most prominent signal intensity. Osteonecrosis of jaw, a rare but serious AE, was considered highly associated with letrozole and exemestane monotherapy in our study (letrozole, n = 138, ROR = 4.37; exemestane, n = 23, ROR = 2.39). Furthermore, our research also discovered that letrozole and exemestane exhibited numerous positive signals with high frequencies and large ROR values in the circulatory and lymphatic systems in the blood and lymphatic system disorders, respiratory, thoracic and mediastinal disorders, hepatobiliary disorders, and other SOCs, some of which were rare but serious AEs, belonging to the category of serious adverse reactions and posing a threat to the patients’ lives.

### 4.1. Joint and Musculoskeletal Toxicity

In the musculoskeletal and connective tissue disorders system, our study found that letrozole, anastrozole and exemestane had 16, 21 and 12 positive signals, respectively, which were reported in ≥30 case reports in the database. The joint and musculoskeletal toxicity profiles of the three AIs were much similar. A wide range of musculoskeletal toxicity occurrences, with manifestations including trigger finger, bone pain, osteopenia, arthralgia, joint stiffness, myalgia, osteoarthritis, and so on, were reported in long-term side effects of the three AIs. Estrogen exhibits a crucial function in sustaining the normal bone mass of human skeleton by balancing the dynamic process of bone resorption by osteoclasts and bone formation by osteoblasts, in which estrogen is the main steroid that participates in bone resorption [22,23]. Estrogen deprivation that occurs in gonads and extra-gonadal organs under the treatment of AIs is generally abrupt, which reduces the favorable effects of estrogen on bone health and shifts the balance of bone remodeling toward increased osteoclasts-mediated bone resorption, resulting in net bone loss and decreased bone mass [22,23]. Earlier researches have indicated that the bone mineral density in women after menopause declined by 1.1% to 3.4% within the initial year of AI therapy, which could surpass the annual bone loss rate of 1% to 2% during menopause [23]. A large retrospective cohort study divided more than 12,000 patients with non-metastatic BC into three groups based on receiving AI treatment or anti-estrogen therapy or no medication, and found a significant association between AI therapy and a higher likelihood of suffering bone loss and fractures [23]. A twofold risk of pathological fracture occurs when there is a 10% to 15% decline in bone mineral density [23]. As expected, in our study, there were 32 cases of pathological fractures (ROR = 5.30), 45 cases of humerus fractures (ROR = 7.67), and 40 cases of femoral neck fractures (ROR = 6.62) with letrozole. Likewise, 33 cases of wrist fractures (ROR = 5.64) and 32 cases of hip fractures (ROR = 2.08) were recorded in case reports with the use of anastrozole. The concern regarding accelerated osteoporotic fractures generated by AIs is now being gradually addressed through the use of bisphosphonates [24]. One proposed theory has shown that exemestane as a steroidal AI had fewer AEs related to bone health than anastrozole and letrozole. The researchers speculated that its bone protective effect might be attributed to the androgenic properties of its principal metabolite, 17-hydroexemestane [23]. However, further research is needed to ascertain whether there is a disparity in the rate of bone loss among the three AIs, and whether this difference exists between nonsteroidal AI and steroidal AI (a class drug effect) or among the three AIs (an individual drug effect).

The musculoskeletal pain of AIs has been widely regarded as concerning. Arthralgia is one of the most common complications of musculoskeletal pain in AI-treated patients. Symmetric joint pain and stiffness, commonly affecting the hands, wrists, knees, ankles, shoulders, and the central joints of the axial spine and pelvis, are frequently observed in AI-induced arthralgia [25,26]. A meta-analysis encompassing 13,000 individuals revealed that the occurrence rate of arthralgia in women treated with AI was between 20–74% [27]. In our study, arthralgia reported the highest frequency of cases for anastrozole and exemestane, and the second highest frequency of cases for letrozole, after neutropenia. Besides, patients with BC treated with AI therapy are also prone to suffer myalgia [28]. In our study, myalgia ranked in the top 10 ordered by reporting frequency for all three AIs. At present, various mechanisms of AI-induced musculoskeletal pain have been proposed. Most scholars have found that aromatase was expressed in synovial cells and chondrocytes of articular cartilage, so a proposed explanation argued that the absence of estrogen caused by AIs promoted the production of pro-inflammatory cytokines such as interleukin-6 (IL-6) and IL-1 in articular chondrocytes, hindering the chondroprotective effect of estrogen, thus leading to joint pain and swelling [29,30]. Compelling evidence suggested that transient receptor potential ankyrin 1 (TRPA1) channel, a major pathway of pain transmission and neurogenic inflammation, might be involved in AI-related pain due to the electrophilicity of AIs [31]. In animal models, AI-induced pain-like symptoms were significantly diminished in mice with TRPA1 pharmacological blockade or TRPA1 deficiency [31]. Kinin is a potent endogenous algogenic mediator contributing to inflammation and pain processes via activating B1 and B2 receptor and prompting these receptors to interact with TRPA1 [32]. A study has found that AIs could increase the expression of kinin receptors, and AI-induced mechanical allodynia and decreased muscle strength in mice were shown to be antagonized by kinin B1 (DALBk) and B2 (Icatibant) receptor inhibitors [32]. Other studies have found that single-nucleotide polymorphisms in certain genes are involved in the development of AI-induced arthralgia and myalgia, such as the rs2073618 variant (G1181C) of the osteoprotegerin (OPG) gene [33,34,35,36]. Medication-induced tendinopathy is also one of the most common complications of AIs. Trigger finger, also sometimes called stenosing flexor tenosynovitis, is a form of tenosynovitis. Our results have revealed that trigger finger was the strongest signal strength for the three AIs, which might be normally linked to the increase responsiveness of inflammatory cells as estrogen production declined [37,38]. However, the exact mechanism of trigger finger induced by AIs is unknown and still needs to be explored. In 2015, a report stated that a 49-year-old female, without any underlying conditions, experienced the emergence of bilateral trigger thumbs in both hands after receiving two years of anastrozole treatment for endometrial adenocarcinoma metastases [39]. According to the result of a single-center, retrospective analysis of BC patients, it was found that patients treated with AIs had a considerably higher likelihood of experiencing trigger fingers in comparison to those not receiving AI therapy (5.1% vs. 1.5%, *p* < 0.001) [40]. Moreover, another retrospective analysis for BC patients figured out a higher incidence of trigger finger induced by the representative agents of hormonal therapy, third-generation AIs, than trastuzumab or tamoxifen, despite them all belonging to breast conservation therapy [38]. Notably, several literatures indicated that most BC patients reported continuous, severe, and debilitating musculoskeletal pains after receiving AI treatment, and a larger rate of these patients reported impaired ability in daily function, poor patient quality of life and treatment adherence, and a higher likelihood of requiring steroid injections and surgical release of trigger finger [38,41].

Osteonecrosis of the jaw, a kind of metabolic disorder and osteonecrosis disease, is a rare drug-related AE, which manifests local redness, pain, fistula formation, jaw necrosis, and dead bone exposure, etc., leading to pathological fractures in severe cases and seriously affecting the patients’ daily functions [22,42]. In our study, we confirmed the occurrence of medication-induced osteonecrosis of the jaw during the administration of letrozole and exemestane, while patients with anastrozole therapy only reported four cases accompanied by a low ROR value. Still, the AE of osteonecrosis of the jaw acted as a cautionary signal that should not be underestimated.

### 4.2. Hematologic Toxicity

No dose-related effect on any hematologic parameter was evident in the instruction of letrozole. Besides, hematologic toxicity induced by anastrozole or exemestane was not mentioned in their instructions. Hematological events potentially associated with AIs were first documented in our research. We found that the rare and severe hematologic toxicity of letrozole was more frequent than that of anastrozole and exemestane, which included neutropenia (n = 1843, ROR = 12.49), neutrophil count decreased (n = 182, ROR = 4.03), leukopenia (n = 635, ROR = 11.11), white blood cell count decreased (n = 427, ROR = 12.49), thrombocytopenia (n = 527, ROR = 4.09), red blood cell count decreased (n = 78, ROR = 2.30), lymphopenia (n = 66, ROR = 4.08), lymphocyte count decreased (n = 44, ROR = 2.07), anaemia (n = 674, ROR = 2.94), myelosuppression (n = 45, ROR = 2.07), and so on. Most notably, neutropenia was the highest-frequency AE for letrozole in the FAERS database. Furthermore, the predominant grade 3 or 4 AE related to hematologic toxicity caused by exemestane was neutropenia (n = 97, ROR = 2.09), followed by leukopenia (n = 67, ROR = 3.80). Interestingly, our study did not detect anastrozole-associated positive signal AEs in the hematologic system. Although the clinical significance of AIs-related hematologic toxicity is uncertain due to lack of literature support, patients should be more alert to the occurrence of abnormal hematologic events.

### 4.3. Respiratory Toxicity

Our study found that letrozole had a significant impact on the respiratory system, with pneumonitis (n = 109, ROR = 3.70), ILD (n = 159, ROR = 2.91), PE (n = 272, ROR = 2.30) and atelectasis (n = 30, ROR = 2.47) exhibiting high frequency and large ROR values. Similar AEs for respiratory toxicity occurred in exemestane, which included pneumonitis (n = 67, ROR = 7.53), ILD (n = 72, ROR = 4.36), and PE (n = 82, ROR = 2.29), while anastrozole was associated with fewer respiratory side effects and ILD had the highest frequency and the strongest signal intensity (n = 47, ROR = 2.10). Severe respiratory side effects associated with AIs, such as ILD and PE, were not mentioned in the instruction but were clinically important. Our research found that ILD occurred in all three drugs; however, there were few literatures on AI-related ILD. A 72-year-old woman who was receiving antiestrogen therapy with AIs suffered the development of interstitial pneumonitis, as described in a case report published in 2020. Although the patient tried multiple AI monotherapy, switching from anastrozole to letrozole and finally to exemestane, her lung lesions recurred as soon as the drug was initiated [43]. ILD is a potentially life-threatening AE, and permanent discontinuation is recommended once grade ≥ 2 ILD happens. Besides, the AE of PE should not be underestimated. In 2021, An 87-year-old woman receiving anastrozole therapy was reported to have diffuse venous thromboembolism in a case. PE occurred when blood clots dislodged from the veins and traveled to her lungs, causing chest pain and breathing difficulties [44]. In 2004, a case report described that a 72-year-old female patient with postmenopausal stage IIB disease developed PE after switching to letrozole due to a severe deep venous thrombosis on adjuvant tamoxifen [45]. Another case reported that an 80-year-old woman suffered from acute bilateral PE after about 2 years of anastrozole therapy [46]. PE ranks as the third leading cause of cardiovascular fatality after stroke and myocardial infarction [47]. Monitoring coagulation system function is highly advised before starting and throughout the course of treatment. 

### 4.4. Hepatic Toxicity

Over the last few years, some cases of drug-induced hepatotoxicity secondary to AIs have been documented. In 2017, A case of letrozole-induced hepatotoxicity was published. The patient aged 70 years old was reported to have acute hepatitis with a sudden increase of hepatic transaminases and abnormal histological features displayed by liver biopsy [48]. Besides, an in vivo experiment showed that letrozole increased hepatic function parameters such as aspartate transaminase, lactate dehydrogenase, alkaline phosphatase, and bilirubin [49]. Consistently, in our study, we found that similar increased liver enzymes were reported for letrozole in FAERS (Table S9). Likewise, a case of severe prolonged cholestatic hepatitis induced by exemestane was reported to occur in a 47-year-old female [50]. Anastrozole, on the other hand, has been generally considered as a good liver safety profile in clinical trials [51]. Consistently, in our study, we found that anastrozole-associated hepatotoxicity was rare in the FAERS database; however, some reported cases occurring suggested a probable correlation between anastrozole and liver damage. An 89-year-old woman with invasive lobular BC was reported to develop acute mixed but primarily cholestatic hepatic damage after 2 months of treatment with anastrozole [52]. The patient mainly manifested mild conjunctival jaundice and a transient elevation of hepatic enzymes, but recovered promptly and fully after drug withdrawal. A patient aged 58 years old also suffered from similar cholestatic liver damage during anastrozole therapy [53]. The third case reported that a 70-year-old woman developed acute hepatitis with increased hepatic enzymes and positive serum antinuclear antibodies due to the treatment of anastrozole [54]. The biopsy findings showed mild hepatic steatosis, moderate levels of inflammation, and moderate to severe fibrosis in liver.

At present, the mechanism of AIs-associated hepatotoxicity is still unclear. Some researchers linked the histological localization of AIs-induced hepatocyte necrosis to the proposed hypothesis of metabolism-mediated hepatocellular liver injury [53,54,55]. In fact, the genetic polymorphism of any drug metabolism enzymes could predispose to hepatotoxicity secondary to AIs [53,54]. Given that the toxic potential of pharmacologically inactive metabolites of AIs remains uncertain, other risks, such as the immune-mediated mechanism and individual susceptibility, cannot be excluded. So far, AIs have been used as a standard first-line adjuvant therapy for postmenopausal BC patients with positive hormone receptor. These patient populations are generally apt to receive AIs administration for up to 10 years [48]. Thus, AI-induced hepatotoxicity is very likely to occur, and this rare but life-threatening complication must be monitored closely during the first 6 months, followed by regular follow-up examinations [48].

### 4.5. Limitations

There are, in addition, limitations to the study that should be noted. Firstly, the FAERS database is a self-reporting system, receiving voluntary reports from the public of different ages, backgrounds, and professions, which may have some inherent shortcomings, such as underreporting, false, and inaccurate reporting, leading to reporting bias to some extent. Secondly, the information on indication/diagnosis is incomplete and unspecific in the INDI files in the FAERS database, such as the data about the type and the stage of BC, which is insufficient to support an in-depth and extended analysis. Thirdly, the clinical priority of endocrine therapeutic drugs for BC patients at earlier stages is yet to be assessed. The signal mining and analysis of AE signals induced by other drugs parallel to AIs, such as tamoxifen, and the combination of tamoxifen and AIs, is conducive to improving the risk awareness of AEs and obtaining a more comprehensive safety assessment. Last but not least, only a statistical correlation between an AI and certain AE is proven by signal detection. There is no sufficient evidence to support a necessary causal relationship, which still needs to be further studied and evaluated in combination with relevant literatures and clinical studies.

## 5. Conclusions

Based on the extensive real-world data from the FAERS database, our study analyzed the treatment safety of letrozole, anastrozole and exemestane, and found the result of AEs for these three AIs associated with the musculoskeletal and connective tissue disorders system, which basically agreed with the result in the drug instruction, suggesting the reliability of the FAERS database and the data mining method used herein. The result also suggested that all three AIs could cause ILD, a rare but serious AE. In addition, this study also revealed that letrozole might also have many rare but serious AEs with high frequency or strong ROR or not mentioned in the manual, such as neutropenia, leukopenia, polyneuropathy, hypersensitivity vasculitis, thrombocytopenia, PE, osteonecrosis of jaw, and so on. Overall, this study is helpful to improve the awareness of clinicians and pharmacists on the risk signals related to third-generation AIs, and to take preventive measures in time to ensure the safety of drug use.

## Figures and Tables

**Table 1:** Clinical characteristics of patients treated with AIs from the FAERS database.

Characteristics		Patients, N (%)		
		Letrozole	Anastrozole	Exemestane
Gender	Male	208 (1.15%)	126 (1.53%)	49 (0.70%)
	Female	16,423 (91.06%)	7911 (95.98%)	6467 (92.24%)
	Unknown	1404 (7.78%)	205 (2.49%)	495 (7.06%)
Age (year)	<18	48 (0.27%)	22 (0.27%)	2 (0.03%)
	18–64	5181 (28.73%)	2316 (28.10%)	2034 (29.01%)
	65–85	4536 (25.15%)	2761 (33.50%)	2743 (39.12%)
	>85	321 (1.78%)	209 (2.54%)	226 (3.22%)
	Unknown	7949 (44.08%)	2934 (35.60%)	2006 (28.61%)
Weight (kg)	<50	364 (2.02%)	157 (1.90%)	88 (1.26%)
	50–100	4730 (26.23%)	2734 (33.17%)	2064 (29.44%)
	>100	335 (1.86%)	193 (2.34%)	166 (2.37%)
	Unknown	12,606 (69.90%)	5158 (62.58%)	4693 (66.94%)
Reporter	Medical professional	11,716 (64.96%)	2604 (31.59%)	3590 (51.21%)
	Non-medical professional	5509 (30.55%)	3846 (46.66%)	3158 (45.04%)
	Unknown	810 (4.49%)	1792 (21.74%)	263 (3.75%)
Reporter’s country	USA	4617 (25.60%)	5997 (72.76%)	4029 (57.47%)
	Others	13,418 (74.40%)	2245 (27.24%)	2982 (42.53%)
Outcome of AEs	Hospitalization	4519 (21.00%)	1294 (14.55%)	1389 (17.53%)
	Disability	552 (2.57%)	324 (3.64%)	168 (2.12%)
	Life-threatening	658 (3.06%)	136 (1.53%)	169 (2.13%)
	Death	1484 (6.90%)	369 (4.15%)	685 (8.65%)
	Congenital anomaly	108 (0.50%)	3 (0.03%)	2 (0.03%)
	Permanent impairment/damage	26 (0.12%)	128 (1.44%)	13 (0.16%)
	Others	14,171 (65.86%)	6641 (74.66%)	5496 (69.38%)

Abbreviations: AI: aromatase inhibitor; AE: adverse event; USA: the United States of America.

**Table 2:** WSP test for AEs related to letrozole, anastrozole, and exemestane.

Drug	Patients (N)	Time-to-Onset (Median, IQR)	Scale Parameter: α (95% CI)	Shape Parameter: β (95% CI)	Type
Letrozole	5228	99.50 (IQR 25.00–431.00)	227.57 (217.32–237.81)	0.64 (0.62–0.65)	Early failure
Anastrozole	1999	268.00 (IQR 61.00–791.00) ^a,b^	433.43 (405.21–461.65)	0.71 (0.68–0.73)	Early failure
Exemestane	1533	116.00 (IQR 31.00–437.00) ^c^	250.66 (231.07–270.25)	0.68 (0.65–0.70)	Early failure

^a^: Letrozole vs. Anastrozole, *p* < 0.0001; ^b^: Anastrozole vs. Exemestane, *p* < 0.0001; ^c^: Letrozole vs. Exemestane, *p* = 0.019. Abbreviations: WSP: Weibull shape parameter; AE: adverse event; IQR: interquartile range; CI: confidence interval.

## Data Availability

The dataset from FAERS database generated and/or analyzed during the current study are available in the database dataset repository (https://fis.fda.gov/extensions/FPD-QDE-FAERS/FPD-QDE-FAERS.html, accessed on 4 June 2024).

## Supplementary Materials

The following supporting information can be downloaded at: https://www.mdpi.com/article/10.3390/biomedicines12081708/s1, Table S1. Two-by-two contingency table for disproportionality analyses. Table S2. Summary of major algorithms used for signal detection. Table S3. The top 30 AEs with the strongest signal intensity for letrozole. Table S4. The top 30 AEs with the strongest signal intensity for anastrozole. Table S5. The top 30 AEs with the strongest signal intensity for exemestane. Table S6. The top 30 AEs with the highest frequency for letrozole. Table S7. The top 30 AEs with the highest frequency for anastrozole. Table S8. The top 30 AEs with the highest frequency for exemestane. Table S9. Letrozole-related positive AE reports in each SOC and their signal detection. Table S10. Anastrozole-related positive AE reports in each SOC and their signal detection. Table S11. Exemestane-related positive AE reports in each SOC and their signal detection. Figure S1. The number of involving system signals for three AIs. Figure S2. Subgroup analysis of letrozole use showing the forest plot of ROR (95% CI) for the top 10 AEs with the strongest signal intensity. Figure S3. Subgroup analysis of letrozole use showing the forest plot of ROR (95% CI) for the top 10 AEs with the highest frequency. Figure S4. Subgroup analysis of anastrozole use showing the forest plot of ROR (95% CI) for the top 10 AEs with the strongest signal intensity. Figure S5. Subgroup analysis of anastrozole use showing the forest plot of ROR (95% CI) for the top 10 AEs with the highest frequency. Figure S6. Subgroup analysis of exemestane use showing the forest plot of ROR (95% CI) for the top 10 AEs with the strongest signal intensity. Figure S7. Subgroup analysis of exemestane use showing the forest plot of ROR (95% CI) for the top 10 AEs with the highest frequency.
