# Supplementary material for: Adverse Event Profiles of the Third-Generation Aromatase Inhibitors: Analysis of Spontaneous Reports Submitted to FAERS
Source: Biomedicines. 2024 Aug 1;12(8):1708. doi: 10.3390/biomedicines12081708 (PMC11351598; doi:10.3390/biomedicines12081708)
Supplement: Supplementary file 1 [file biomedicines-12-01708-s001.zip › biomedicines-3069568-supplementary.pdf]

Supporting Information

Adverse Event Profiles of the Third-Generation Aromatase Inhibitors: Analysis of Spontaneous Reports Submitted to FAERS

Yina Zhang, Lingzhu Zhao, Yanning Liu, Jingkang Zhang, Luyan Zheng and Min Zheng *

State Key Laboratory for Diagnosis and Treatment of Infectious Diseases, National Clinical Research Center for Infectious Diseases, Collaborative Innovation Center for Diagnosis and Treatment of Infectious Diseases, The First Affiliated Hospital, College of Medicine, Zhejiang University, 79 Qingchun Road, Hangzhou 310003, China

* Correspondence: minzheng@zju.edu.cn

Supplementary materials:

Table S1. Two-by-two contingency table for disproportionality analyses.

	AEs of interest	Non-interested AEs	Total
Drug of interest	a	b	a+b
Non-interested drug	c	d	c+d
Total	a+c	b+d	a+b+c+d

Abbreviations: AE: adverse event.

Table S2. Summary of major algorithms used for signal detection.

Algorithms	Equation	Criteria
ROR	ROR=ad/bc	95% CI > 1，N≥3
	95% CI=$e^{\ln(ROR)\pm1.96(1/a+1/b+1/c+1/d)^{\wedge}0.5}$	
PRR	PRR=(a/(a+b))/(c/(c+d))	PRR≥2，χ^2≥4，N≥3
	χ^2=[(ad-bc)^2] (a+b+c+d)/	
	[(a+b)(c+d)(a+c)(b+d)]	

Abbreviations: ROR: reporting odds ratio; CI: confidence interval; RRR: proportional reporting ratio.

Table S3. The top 30 AEs with the strongest signal intensity for letrozole.

Top 30	AE	n	PRR（χ^2）	ROR（95% CI）
1	Trigger finger	112	24.97 (2488.46)	25.01 (20.71–30.20)
2	Skin hypopigmentation	51	24.87 (1128.03)	24.88 (18.82–32.90)
3	Bone lesion	76	18.04 (1192.62)	18.06 (14.38–22.68)
4	Uterine polyp	40	19.60 (686.71)	19.61 (14.32–26.85)
5	Vulvovaginal dryness	61	16.21 (850.75)	16.22 (12.58–20.91)
6	Mucosal dryness	39	16.67 (561.15)	16.68 (12.14–22.92)
7	Neutropenia	1843	12.20 (18657.99)	12.49 (11.92–13.09)
8	Leukopenia	635	11.02 (5700.75)	11.11 (10.27–12.02)
9	Hepatic lesion	60	12.09 (600.24)	12.10 (9.38–15.62)
10	Polyneuropathy	140	10.67 (1209.01)	10.69 (9.05–12.64)
11	Bone pain	535	7.67 (3074.48)	7.72 (7.09–8.41)
12	Hot flush	616	7.51 (3442.17)	7.56 (6.98–8.19)
13	Hypersensitivity vasculitis	33	9.21 (238.40)	9.21 (6.54–12.99)
14	Onychomadesis	32	8.574 (211.53)	8.58 (6.05–12.16)
15	Gamma-glutamyltransferase increased	194	6.93 (974.93)	6.94 (6.03–8.00)
16	Hepatotoxicity	172	6.88 (855.93)	6.89 (5.93–8.01)
17	Humerus fracture	45	7.67 (258.01)	7.67 (5.72–10.29)
18	Erysipelas	45	7.52 (251.55)	7.52 (5.61–10.09)
19	Electrocardiogram qt prolonged	247	5.79 (971.75)	5.81 (5.12–6.58)
20	Hepatic cytolysis	75	6.29 (330.67)	6.29 (5.01–7.90)
21	Nail disorder	57	6.32 (253.07)	6.33 (4.87–8.21)
22	Femoral neck fracture	40	6.62 (189.12)	6.62 (4.85–9.05)
23	Onychoclasis	51	6.35 (227.99)	6.36 (4.82–8.38)

24	Skin toxicity	35	6.15 (149.68)	6.15 (4.41–8.58)
25	Organising pneumonia	35	6.12 (148.54)	6.12 (4.39–8.54)
26	Hydronephrosis	51	5.72 (197.07)	5.72 (4.34–7.54)
27	Carpal tunnel syndrome	89	5.32 (309.92)	5.33 (4.32–6.56)
28	Spinal pain	73	5.32 (254.16)	5.32 (4.23–6.70)
29	Thrombocytopenia	527	4.07 (1213.94)	4.09 (3.75–4.46)
30	Pathological fracture	32	5.29 (110.64)	5.30 (3.74–7.50)

Abbreviations: AE: adverse event; ROR: reporting odds ratio; CI: confidence interval; RRR: proportional reporting ratio.

Table S4. The top 30 AEs with the strongest signal intensity for anastrozole.

Top 30	AE	n	PRR (χ^2)	ROR (95% CI)
1	Trigger finger	131	71.93 (8792.87)	72.25 (60.64–86.08)
2	Vulvovaginal dryness	87	57.15 (4644.25)	57.31 (46.28–70.99)
3	Hot flush	710	21.22 (13525.56)	21.71 (20.15–23.40)
4	Carpal tunnel syndrome	121	17.75 (1893.63)	17.82 (14.89–21.32)
5	Osteopenia	117	15.08 (1525.16)	15.14 (12.61–18.16)
6	Bone pain	353	12.35 (3658.32)	12.48 (11.24–13.87)
7	Hair growth abnormal	50	14.79 (637.23)	14.81 (11.21–19.57)
8	Laryngeal pain	47	14.23 (573.36)	14.25 (10.69–18.99)
9	Body height decreased	72	12.52 (758.08)	12.55 (9.95–15.83)
10	Onychoclasis	40	12.17 (407.33)	12.19 (8.93–16.64)
11	Nail disorder	44	11.92 (437.13)	11.94 (8.87–16.06)
12	Arthritis	341	8.95 (2398.14)	9.04 (8.12–10.06)
13	Osteoporosis	178	8.69 (1206.17)	8.74 (7.54–10.13)
14	Joint stiffness	104	8.14 (649.03)	8.17 (6.74–9.91)
15	Arthralgia	1215	6.23 (5351.60)	6.45 (6.09–6.84)
16	Blood cholesterol increased	151	6.82 (747.39)	6.85 (5.83–8.04)
17	Tendonitis	56	7.11 (293.08)	7.13 (5.48–9.27)
18	Bone disorder	65	6.74 (316.73)	6.75 (5.29–8.62)
19	Mood altered	82	6.24 (359.32)	6.25 (5.03–7.77)
20	Night sweats	90	6.07 (380.27)	6.09 (4.95–7.49)
21	Bone density decreased	96	5.74 (374.85)	5.76 (4.71–7.04)
22	Ill-defined disorder	139	4.99 (442.44)	5.01 (4.24–5.92)
23	Osteoarthritis	99	4.87 (304.26)	4.89 (4.01–5.96)
24	Wrist fracture	33	5.64 (125.48)	5.64 (4.01–7.94)
25	Mood swings	73	4.49 (197.47)	4.50 (3.57–5.66)
26	Arthropathy	114	4.22 (279.57)	4.23 (3.52–5.09)
27	Myalgia	315	3.80 (649.99)	3.83 (3.43–4.28)
28	Alopecia	356	3.65 (686.14)	3.68 (3.32–4.09)
29	Weight increased	387	3.63 (739.48)	3.67 (3.32–4.05)
30	Pain in extremity	462	3.12 (666.96)	3.15 (2.87–3.46)

Abbreviations: AE: adverse event; ROR: reporting odds ratio; CI: confidence interval; RRR: proportional reporting ratio.

Table S5. The top 30 AEs with the strongest signal intensity for exemestane.

Top 30	AE	n	PRR (χ^2)	ROR (95% CI)
1	Trigger finger	37	26.66 (903.55)	26.71 (19.31–36.94)
2	Hot flush	261	10.46 (2226.43)	10.58 (9.36–11.95)
3	Bone pain	190	8.96 (1338.94)	9.03 (7.82–10.41)
4	Carpal tunnel syndrome	44	8.67 (297.55)	8.69 (6.46–11.68)
5	Pneumonitis	67	7.51 (376.94)	7.53 (5.92–9.57)
6	Osteopenia	42	7.28 (226.81)	7.29 (5.38–9.87)
7	Mucosal inflammation	53	5.78 (208.93)	5.79 (4.42–7.58)
8	Stomatitis	103	4.89 (318.38)	4.91 (4.04–5.96)
9	Arthralgia	606	4.19 (1481.37)	4.29 (3.95–4.65)
10	Gamma-glutamyltransferase increased	45	5.28 (155.78)	5.29 (3.95–7.09)
11	Blood alkaline phosphatase increased	47	4.91 (145.96)	4.92 (3.69–6.55)
12	Bone disorder	36	5.04 (116.37)	5.05 (3.64–7.00)
13	Interstitial lung disease	72	4.34 (185.18)	4.36 (3.46–5.49)
14	Joint stiffness	40	4.22 (98.26)	4.23 (3.10–5.77)
15	Leukopenia	67	3.79 (137.80)	3.80 (2.99–4.84)
16	Alopecia	226	3.13 (328.54)	3.15 (2.77–3.60)
17	Hypoacusis	60	3.48 (106.03)	3.49 (2.71–4.49)
18	Transaminases increased	31	3.84 (64.98)	3.84 (2.70–5.47)
19	Myalgia	179	2.92 (225.71)	2.93 (2.53–3.40)
20	Arthritis	88	3.11 (125.99)	3.12 (2.53–3.85)
21	Hepatic function abnormal	43	3.38 (71.97)	3.38 (2.51–4.57)
22	Osteoporosis	48	3.16 (70.69)	3.16 (2.38–4.20)
23	Night sweats	35	3.19 (52.47)	3.19 (2.29–4.45)
24	Arthropathy	58	2.90 (72.12)	2.90 (2.24–3.76)
25	Vaginal haemorrhage	48	2.92 (60.68)	2.93 (2.20–3.89)
26	Liver function test abnormal	35	3.07 (48.67)	3.07 (2.20–4.28)
27	Diabetes mellitus	77	2.69 (81.70)	2.69 (2.15–3.37)
28	Aspartate aminotransferase increased	53	2.68 (55.92)	2.69 (2.05–3.52)
29	Cataract	53	2.62 (53.17)	2.63 (2.01–3.44)
30	Hepatic failure	31	2.78 (35.26)	2.78 (1.95–3.96)

Abbreviations: AE: adverse event; ROR: reporting odds ratio; CI: confidence interval; RRR: proportional reporting ratio.

Table S6. The top 30 AEs with the highest frequency for letrozole.

Top 30	AE	n	PRR（χ^2）	ROR（95% CI）
1	Neutropenia	1843	12.20 (18657.99)	12.49 (11.92–13.09)
2	Arthralgia	1341	2.81 (1568.38)	2.84 (2.69–3.00)
3	Alopecia	706	2.96 (918.23)	2.98 (2.77–3.21)
4	Anaemia	674	2.92 (852.95)	2.94 (2.73–3.18)
5	Leukopenia	635	11.02 (5700.75)	11.11 (10.27–12.02)
6	Hot flush	616	7.51 (3442.17)	7.56 (6.98–8.19)
7	Bone pain	535	7.67 (3074.48)	7.72 (7.09–8.41)
8	Thrombocytopenia	527	4.07 (1213.94)	4.09 (3.75–4.46)
9	Myalgia	466	2.30 (341.70)	2.31 (2.11–2.53)
10	White blood cell count decreased	427	3.34 (699.19)	3.36 (3.05–3.69)
11	Pulmonary embolism	272	2.29 (197.54)	2.30 (2.04–2.59)
12	Alanine aminotransferase increased	264	3.51 (471.55)	3.52 (3.12–3.97)
13	Neuropathy peripheral	257	2.38 (205.67)	2.39 (2.11–2.70)
14	Aspartate aminotransferase increased	248	3.81 (511.45)	3.82 (3.37–4.33)
15	Electrocardiogram qt prolonged	247	5.79 (971.75)	5.81 (5.12–6.58)
16	Blood creatinine increased	241	3.01 (323.09)	3.029 (2.66–3.43)
17	Gamma-glutamyltransferase increased	194	6.93 (974.93)	6.94 (6.03–8.00)
18	Neutrophil count decreased	182	4.02 (410.44)	4.03 (3.48–4.66)
19	Hepatotoxicity	172	6.88 (855.93)	6.89 (5.93–8.01)
20	Sleep disorder	159	2.03 (83.25)	2.03 (1.74–2.38)
21	Interstitial lung disease	159	2.91 (198.04)	2.91 (2.49–3.40)
22	Osteoporosis	151	3.01 (201.91)	3.01 (2.57–3.54)
23	Polyneuropathy	140	10.67 (1209.00)	10.69 (9.05–12.64)
24	Stomatitis	140	2.01 (70.91)	2.01 (1.70–2.38)
25	Osteonecrosis of jaw	138	4.36 (355.15)	4.37 (3.69–5.16)
26	Lymphadenopathy	135	3.21 (204.81)	3.22 (2.72–3.81)
27	Blood alkaline phosphatase increased	134	4.24 (330.41)	4.25 (3.59–5.04)
28	Mucosal inflammation	123	4.06 (282.68)	4.07 (3.41–4.86)
29	Joint stiffness	117	3.75 (234.34)	3.75 (3.13–4.50)
30	Hypokalaemia	115	2.14 (69.46)	2.14 (1.78–2.57)

Abbreviations: AE: adverse event; ROR: reporting odds ratio; CI: confidence interval; RRR: proportional reporting ratio.

Table S7. The top 30 AEs with the highest frequency for anastrozole.

Top 30	AE	n	PRR（χ^2）	ROR（95% CI）
1	Arthralgia	1215	6.23 (5351.60)	6.45 (6.09–6.84)
2	Hot flush	710	21.22 (13525.56)	21.71 (20.15–23.40)
3	Pain in extremity	462	3.12 (666.96)	3.15 (2.87–3.46)
4	Weight increased	387	3.63 (739.48)	3.67 (3.32–4.05)
5	Insomnia	376	2.85 (451.73)	2.87 (2.59–3.18)
6	Alopecia	356	3.65 (686.14)	3.68 (3.32–4.09)
7	Bone pain	353	12.35 (3658.32)	12.48 (11.24–13.87)
8	Arthritis	341	8.95 (2398.14)	9.04 (8.12–10.06)
9	Myalgia	315	3.80 (649.99)	3.83 (3.43–4.28)
10	Depression	304	2.62 (305.71)	2.64 (2.36–2.95)
11	Back pain	246	2.16 (152.72)	2.16 (1.91–2.45)
12	Gait disturbance	211	2.18 (134.23)	2.18 (1.91–2.50)
13	Hypoaesthesia	203	2.72 (221.15)	2.73 (2.38–3.14)
14	Osteoporosis	178	8.69 (1206.17)	8.74 (7.54–10.13)
15	Paraesthesia	170	2.15 (104.35)	2.15 (1.85–2.50)
16	Blood cholesterol increased	151	6.82 (747.39)	6.85 (5.83–8.04)
17	Musculoskeletal stiffness	142	3.32 (230.57)	3.33 (2.83–3.93)
18	Ill-defined disorder	139	4.99 (442.44)	5.01 (4.24–5.92)
19	Neuropathy peripheral	136	3.08 (191.02)	3.08 (2.61–3.66)
20	Muscular weakness	133	2.41 (109.43)	2.41 (2.04–2.86)
21	Trigger finger	131	71.93 (8792.87)	72.25 (60.64–86.08)
22	Carpal tunnel syndrome	121	17.75 (1893.63)	17.82 (14.89–21.32)
23	Joint swelling	118	2.07 (65.74)	2.08 (1.73–2.49)
24	Osteopenia	117	15.08 (1525.16)	15.14 (12.61–18.16)
25	Arthropathy	114	4.22 (279.57)	4.23 (3.52–5.09)
26	Joint stiffness	104	8.14 (649.03)	8.17 (6.74–9.91)
27	Osteoarthritis	99	4.87 (304.26)	4.89 (4.01–5.96)
28	Musculoskeletal pain	98	3.47 (172.27)	3.48 (2.85–4.24)
29	Adverse drug reaction	97	2.45 (83.18)	2.45 (2.01–3.00)
30	Bone density decreased	96	5.74 (374.85)	5.76 (4.716–7.04)

Abbreviations: AE: adverse event; ROR: reporting odds ratio; CI: confidence interval; RRR: proportional reporting ratio.

Table S8. The top 30 AEs with the highest frequency for exemestane.

Top 30	AE	n	PRR（χ^2）	ROR（95% CI）
1	Arthralgia	606	4.19 (1481.37)	4.29 (3.95–4.65)
2	Malaise	327	2.02 (168.49)	2.03 (1.82–2.27)
3	Hot flush	261	10.46 (2226.43)	10.58 (9.36–11.95)
4	Alopecia	226	3.13 (328.54)	3.15 (2.77–3.60)
5	Insomnia	215	2.20 (141.12)	2.21 (1.93–2.53)
6	Bone pain	190	8.96 (1338.94)	9.03 (7.82–10.41)
7	Myalgia	179	2.92 (225.71)	2.93 (2.53–3.40)
8	Stomatitis	103	4.89 (318.38)	4.91 (4.04–5.96)
9	Neutropenia	97	2.09 (55.20)	2.09 (1.72–2.56)
10	Arthritis	88	3.11 (125.99)	3.12 (2.53–3.85)
11	Pulmonary embolism	82	2.28 (58.99)	2.29 (1.84–2.84)
12	Diabetes mellitus	77	2.69 (81.70)	2.69 (2.15–3.37)
13	Neuropathy peripheral	73	2.23 (49.77)	2.24 (1.78–2.82)
14	Interstitial lung disease	72	4.34 (185.18)	4.36 (3.46–5.49)
15	Pneumonitis	67	7.51 (376.94)	7.53 (5.92–9.57)
16	Leukopenia	67	3.79 (137.80)	3.80 (2.99–4.84)
17	Hypoacusis	60	3.48 (106.03)	3.49 (2.71–4.49)
18	Arthropathy	58	2.90 (72.12)	2.90 (2.24–3.76)
19	Alanine aminotransferase increased	56	2.45 (48.20)	2.46 (1.89–3.19)
20	Cataract	53	2.62 (53.17)	2.63 (2.01–3.44)
21	Mucosal inflammation	53	5.78 (208.93)	5.79 (4.42–7.58)
22	Aspartate aminotransferase increased	53	2.68 (55.92)	2.69 (2.05–3.52)
23	Musculoskeletal pain	50	2.39 (40.52)	2.40 (1.81–3.16)
24	Sleep disorder	49	2.07 (27.06)	2.07 (1.56–2.74)
25	Osteoporosis	48	3.16 (70.69)	3.16 (2.38–4.20)
26	Vaginal haemorrhage	48	2.92 (60.68)	2.93 (2.20–3.89)
27	Vertigo	47	2.10 (27.08)	2.10 (1.58–2.80)
28	Blood alkaline phosphatase increased	47	4.91 (145.96)	4.92 (3.69–6.55)
29	Gamma-glutamyltransferase increased	45	5.28 (155.78)	5.29 (3.95–7.09)
30	Carpal tunnel syndrome	44	8.67 (297.55)	8.67 (6.46–11.68)

Abbreviations: AE: adverse event; ROR: reporting odds ratio; CI: confidence interval; RRR: proportional reporting ratio.

Table S9. Letrozole-related positive AE reports in each SOC and their signal detection.

SOC system	AE	n	PRR（χ^2）	ROR（95% CI）
Investigations	Gamma-glutamyltransferase increased	194	6.93 (974.93)	6.94 (6.03–8.00)
	Electrocardiogram qt prolonged	247	5.79 (971.75)	5.81 (5.12–6.58)
	Blood alkaline phosphatase increased	134	4.24 (330.41)	4.25 (3.59–5.04)
	Neutrophil count decreased	182	4.02 (410.44)	4.03 (3.48–4.66)
	Aspartate aminotransferase increased	248	3.81 (511.45)	3.82 (3.37–4.33)
	Transaminases increased	106	3.98 (235.43)	3.99 (3.29–4.83)
	Blood lactate dehydrogenase increased	82	4.01 (184.06)	4.01 (3.23–4.98)
	Alanine aminotransferase increased	264	3.51 (471.55)	3.52 (3.12–3.97)
	Ejection fraction decreased	72	3.87 (152.43)	3.87 (3.07–4.88)
	White blood cell count decreased	427	3.34 (699.19)	3.36 (3.05–3.69)
	Blood bilirubin increased	114	3.42 (194.33)	3.42 (2.85–4.12)
	Glomerular filtration rate decreased	46	3.66 (88.46)	3.66 (2.74–4.89)
	Blood creatinine increased	241	3.01 (323.09)	3.02 (2.66–3.43)
	Liver function test increased	71	3.26 (110.87)	3.26 (2.58–4.12)
	C-reactive protein increased	113	2.72 (122.44)	2.72 (2.26–3.27
	Red blood cell count decreased	78	2.29 (56.76)	2.30 (1.84–2.87)
	Lymphocyte count decreased	44	2.07 (24.24)	2.07 (1.54–2.78)
Musculoskeletal and connective tissue disorders	Trigger finger	112	24.97 (2488.46)	25.01 (20.71–30.20)
	Bone lesion	76	18.04 (1192.62)	18.06 (14.38–22.68)
	Bone pain	535	7.67 (3074.48)	7.72 (7.09–8.41)
	Spinal pain	73	5.32 (254.16)	5.32 (4.23–6.70)
	Pathological fracture	32	5.29 (110.64)	5.30 (3.74–7.50)
	Osteonecrosis of jaw	138	4.36 (355.15)	4.37 (3.69–5.16)
	Joint stiffness	117	3.75 (234.34)	3.75 (3.13–4.50)
	Tendonitis	69	3.58 (127.95)	3.59 (2.83–4.54)
	Arthralgia	1341	2.81 (1568.38)	2.84 (2.69–3.00)
	Osteoporosis	151	3.01 (201.91)	3.01 (2.57–3.54)
	Osteopenia	58	3.04 (79.11)	3.04 (2.35–3.94)
	Myalgia	466	2.30 (341.70)	2.31 (2.11–2.53)
	Bone disorder	61	2.58 (59.05)	2.59 (2.01–3.33)
	Musculoskeletal chest pain	50	2.65 (51.31)	2.65 (2.01–3.50)
	Osteoarthritis	100	2.01 (50.77)	2.01 (1.65–2.45)
	Musculoskeletal discomfort	43	2.11 (25.04)	2.11 (1.56–2.85)
Skin and subcutaneous tissue disorders	Skin hypopigmentation	51	24.87 (1128.03)	24.88 (18.82–32.90)
	Hypersensitivity vasculitis	33	9.21 (238.40)	9.21 (6.54–12.99)
	Onychomadesis	32	8.57 (211.53)	8.58 (6.05–12.16)
	Nail disorder	57	6.32 (253.07)	6.33 (4.87–8.21)
	Onychoclasis	51	6.35 (227.99)	6.36 (4.82–8.38)
	Skin toxicity	35	6.15 (149.68)	6.15 (4.41–8.58)
	Alopecia	706	2.96 (918.23)	2.98 (2.77–3.21)
	Dermatitis bullous	31	3.64 (59.11)	3.64 (2.56–5.19)

	Night sweats	86	2.37 (67.96)	2.37 (1.92–2.93)
Hepatobiliary disorders	Hepatic lesion	60	12.09 (600.24)	12.10 (9.38–15.62)
	Hepatotoxicity	172	6.88 (855.93)	6.89 (5.93–8.01)
	Hepatic cytolysis	75	6.29 (330.67)	6.29 (5.01–7.90)
	Hepatocellular injury	58	2.69 (61.16)	2.69 (2.08–3.48)
	Cholestasis	56	2.54 (52.35)	2.55 (1.96–3.31)
	Hepatic steatosis	55	2.51 (49.84)	2.51 (1.93–3.27)
	Hepatic failure	85	2.31 (62.73)	2.31 (1.87–2.86)
	Jaundice	77	2.28 (55.06)	2.28 (1.82–2.85)
	Drug-induced liver injury	66	2.20 (42.98)	2.20 (1.73–2.80)
Blood and lymphatic system disorders	Neutropenia	1843	12.20 (18657.99)	12.49 (11.92–13.09)
	Leukopenia	635	11.02 (5700.75)	11.11 (10.27–12.02)
	Thrombocytopenia	527	4.07 (1213.94)	4.09 (3.75–4.46)
	Lymphopenia	66	4.07 (152.15)	4.08 (3.20–5.19)
	Haematotoxicity	37	3.87 (78.47)	3.88 (2.81–5.35)
	Anaemia	674	2.92 (852.956)	2.94 (2.73–3.18)
	Lymphadenopathy	135	3.21 (204.81)	3.22 (2.72–3.81)
	Myelosuppression	45	2.07 (24.69)	2.07 (1.54–2.77)
Respiratory, thoracic and mediastinal disorders	Organising pneumonia	35	6.12 (148.54)	6.12 (4.39–8.54)
	Pneumonitis	109	3.70 (213.59)	3.70 (3.07–4.47)
	Pulmonary mass	57	3.36 (94.07)	3.36 (2.59–4.36)
	Interstitial lung disease	159	2.91 (198.04)	2.91 (2.49–3.40)
	Pulmonary embolism	272	2.29 (197.54)	2.30 (2.04–2.59)
	Dyspnoea exertional	99	2.34 (75.93)	2.34 (1.92–2.86)
	Atelectasis	30	2.47 (26.18)	2.47 (1.73–3.54)
Nervous system disorders	Polyneuropathy	140	10.67 (1209.01)	10.69 (9.05–12.64)
	Carpal tunnel syndrome	89	5.32 (309.92)	5.33 (4.32–6.56)
	Ischaemic stroke	62	2.86 (74.46)	2.86 (2.23–3.67)
	Neuropathy peripheral	257	2.38 (205.67)	2.39 (2.11–2.70)
	Taste disorder	48	2.70 (51.36)	2.71 (2.04–3.59)
Metabolism and nutrition disorders	Hypercalcaemia	55	3.77 (111.58)	3.78 (2.90–4.92)
	Hypercholesterolaemia	38	3.87 (80.61)	3.88 (2.82–5.33)
	Hypocalcaemia	59	2.68 (62.14)	2.69 (2.08–3.47)
	Hypokalaemia	115	2.14 (69.46)	2.14 (1.78–2.57)
General disorders and administration site conditions	Mucosal dryness	39	16.67 (561.15)	16.68 (12.14–22.92)
	Therapy partial responder	49	4.59 (136.82)	4.60 (3.47–6.09)
	Mucosal inflammation	123	4.06 (282.68)	4.07 (3.41–4.86)
Gastrointestinal disorders	Aphthous ulcer	32	2.83 (37.61)	2.83 (2.00–4.00)
	Stomatitis	140	2.01 (70.91)	2.01 (1.70–2.38)
	Oral discomfort	37	2.22 (24.60)	2.22 (1.60–3.06)
Eye disorders	Dry eye	101	2.10 (58.42)	2.11 (1.73–2.56)
	Lacrimation increased	70	2.14 (42.45)	2.14 (1.69–2.71)
	Macular degeneration	32	2.34 (24.44)	2.34 (1.65–3.31)

Infections and infestations	Erysipelas	45	7.52 (251.55)	7.52 (5.61–10.09)
	Cystitis	79	2.00 (39.69)	2.01 (1.61–2.50)
Vascular disorders	Hot flush	616	7.51 (3442.17)	7.56 (6.98–8.19)
	Hypertensive crisis	59	4.35 (151.37)	4.35 (3.37–5.62)
Reproductive system and breast disorders	Uterine polyp	40	19.60 (686.71)	19.61 (14.32–26.85)
	Vulvovaginal dryness	61	16.21 (850.75)	16.22 (12.58–20.91)
Injury, poisoning and procedural complications	Humerus fracture	45	7.67 (258.01)	7.67 (5.72–10.29)
	Femoral neck fracture	40	6.62 (189.12)	6.62 (4.85–9.05)
Psychiatric disorders	Sleep disorder	159	2.03 (83.25)	2.03 (1.74–2.38)
Cardiac disorders	Pericardial effusion	71	2.66 (73.35)	2.66 (2.11–3.36)
Renal and urinary disorders	Hydronephrosis	51	5.72 (197.07)	5.72 (4.34–7.54)

Abbreviations: AE: adverse event; SOC: system organ class; ROR: reporting odds ratio; CI: confidence interval; RRR: proportional reporting ratio.

Table S10. Anastrozole-related positive AE reports in each SOC and their signal detection.

SOC system	AE	n	PRR（χ^2）	ROR（95% CI）
Musculoskeletal and connective tissue disorders	Trigger finger	131	71.93 (8792.87)	72.25 (60.64–86.08)
	Osteopenia	117	15.08 (1525.16)	15.14 (12.61–18.16)
	Bone pain	353	12.35 (3658.32)	12.48 (11.24–13.87)
	Aarthritis	341	8.95 (2398.14)	9.04 (8.12–10.06)
	Osteoporosis	178	8.69 (1206.17)	8.74 (7.54–10.13)
	Joint stiffness	104	8.14 (649.03)	8.17 (6.74–9.91)
	Arthralgia	1215	6.23 (5351.60)	6.45 (6.09–6.84)
	Tendonitis	56	7.11 (293.08)	7.13 (5.48–9.27)
	Bone disorder	65	6.74 (316.73)	6.75 (5.29–8.62)
	Osteoarthritis	99	4.87 (304.26)	4.89 (4.01–5.96)
	Arthropathy	114	4.22 (279.57)	4.23 (3.52–5.09)
	Myalgia	315	3.80 (649.99)	3.83 (3.43–4.28)
	Pain in extremity	462	3.12 (666.96)	3.15 (2.87–3.46)
	Musculoskeletal pain	98	3.47 (172.27)	3.48 (2.85–4.24)
	Musculoskeletal stiffness	142	3.32 (230.57)	3.33 (2.83–3.93)
	Fibromyalgia	40	3.65 (76.70)	3.65 (2.68–4.98)
	Muscular weakness	133	2.41 (109.43)	2.41 (2.04–2.86)
	Back pain	246	2.16 (152.72)	2.16 (1.91–2.45)
	Limb discomfort	39	2.59 (38.15)	2.60 (1.90–3.55)
	Joint swelling	118	2.07 (65.74)	2.08 (1.73–2.49)
	Neck pain	59	2.18 (37.53)	2.18 (1.69–2.81)
Nervous system disorders	Carpal tunnel syndrome	121	17.75 (1893.63)	17.82 (14.89–21.32)
	Neuropathy peripheral	136	3.08 (191.02)	3.09 (2.61–3.66)
	Hypoaesthesia	203	2.72 (221.15)	2.73 (2.38–3.14)
	Amnesia	80	2.41 (66.24)	2.42 (1.94–3.01)
	Paraesthesia	170	2.15 (104.35)	2.15 (1.85–2.50)
	Dysstasia	30	2.06 (16.24)	2.06 (1.44–2.94)
Skin and subcutaneous tissue disorders	Hair growth abnormal	50	14.79 (637.239)	14.81 (11.21–19.57)
	Onychoclasis	40	12.17 (407.33)	12.19 (8.93–16.64)
	Nail disorder	44	11.92 (437.13)	11.94 (8.87–16.06)
	Night sweats	90	6.07 (380.27)	6.09 (4.95–7.49)
	Alopecia	356	3.65 (686.14)	3.68 (3.32–4.09)
	Skin disorder	34	2.17 (21.53)	2.18 (1.55–3.05)
General disorders and administration site conditions	Ill-defined disorder	139	4.99 (442.44)	5.01 (4.24–5.92
	Adverse drug reaction	97	2.45 (83.18)	2.45 (2.01–3.00)
	Crying	48	2.55 (45.16)	2.55 (1.92–3.39)
	Gait disturbance	211	2.18 (134.23)	2.18 (1.91–2.50)
	Adverse event	93	2.08 (52.46)	2.09 (1.70–2.56)
Investigations	Body height decreased	72	12.52 (758.08)	12.55 (9.95–15.83)
	Blood cholesterol increased	151	6.82 (747.39)	6.85 (5.83–8.04)
	Bone density decreased	96	5.74 (374.85)	5.76 (4.71–7.04)
	Weight increased	387	3.63 (739.48)	3.67 (3.32–4.05)

Psychiatric disorders	Mood altered	82	6.24 (359.32)	6.25 (5.03–7.77)
	Mood swings	73	4.49 (197.47)	4.50 (3.57–5.66)
	Insomnia	376	2.85 (451.73)	2.87 (2.59–3.18)
	Depression	304	2.62 (305.71)	2.64 (2.36–2.95)
Eye disorders	Macular degeneration	34	6.08 (143.94)	6.09 (4.35–8.53)
	Cataract	82	3.00 (109.40)	3.01 (2.42–3.74)
	Dry eye	54	2.75 (60.10)	2.75 (2.11–3.60)
	Visual acuity reduced	39	2.15 (23.93)	2.15 (1.57–2.94)
Respiratory, thoracic and mediastinal disorders	Laryngeal pain	47	14.23 (573.36)	14.25 (10.69–18.99)
	Interstitial lung disease	47	2.10 (26.93)	2.10 (1.58–2.79)
Reproductive system and breast disorders	Vulvovaginal dryness	87	57.15 (4644.25)	57.31 (46.28–70.99)
	Vaginal haemorrhage	58	2.61 (57.69)	2.62 (2.02–3.39)
Injury, poisoning and procedural complications	Wrist fracture	33	5.64 (125.48)	5.64 (4.01–7.94)
	Hip fracture	32	2.08 (18.02)	2.08 (1.47–2.95)
Gastrointestinal disorders	Dry mouth	83	2.16 (51.76)	2.16 (1.74–2.68)
Vascular disorders	Hot flush	710	21.22 (13525.56)	21.71 (20.15–23.40)
Endocrine disorders	Hypothyroidism	31	2.11 (17.99)	2.11 (1.48–3.00)

Abbreviations: AE: adverse event; SOC: system organ class; ROR: reporting odds ratio; CI: confidence interval; RRR: proportional reporting ratio.

Table S11. Exemestane-related positive AE reports in each SOC and their signal detection.

SOC system	AE	n	PRR（χ^2）	ROR（95% CI）
Musculoskeletal and connective tissue disorders	Trigger finger	37	26.66 (903.55)	26.71 (19.31–36.94)
	Bone pain	190	8.96 (1338.94)	9.03 (7.82–10.41)
	Osteopenia	42	7.28 (226.81)	7.29 (5.38–9.87)
	Arthralgia	606	4.19 (1481.37)	4.29 (3.95–4.65)
	Bone disorder	36	5.04 (116.37)	5.05 (3.64–7.00)
	Joint stiffness	40	4.22 (98.26)	4.23 (3.10–5.77)
	Myalgia	179	2.92 (225.71)	2.93 (2.53–3.40)
	Arthritis	88	3.11 (125.99)	3.12 (2.53–3.85)
	Osteoporosis	48	3.16 (70.69)	3.16 (2.38–4.20)
	Arthropathy	58	2.90 (72.12)	2.90 (2.24–3.76)
	Osteoarthritis	38	2.53 (35.00)	2.53 (1.84–3.48)
	Musculoskeletal pain	50	2.39 (40.52)	2.40 (1.81–3.16)
Investigations	Gamma-glutamyltransferase increased	45	5.28 (155.78)	5.29 (3.95–7.09)
	Blood alkaline phosphatase increased	47	4.91 (145.96)	4.92 (3.69–6.55)
	Transaminases increased	31	3.84 (64.98)	3.84 (2.70–5.47)
	Liver function test abnormal	35	3.07 (48.67)	3.07 (2.20–4.28)
	Aspartate aminotransferase increased	53	2.68 (55.92)	2.69 (2.05–3.52)
	Alanine aminotransferase increased	56	2.45 (48.20)	2.46 (1.89–3.19)
	Blood cholesterol increased	36	2.19 (23.31)	2.19 (1.58–3.04)
Nervous system disorders	Carpal tunnel syndrome	44	8.67 (297.55)	8.69 (6.46–11.68)
	Neuropathy peripheral	73	2.23 (49.77)	2.24 (1.78–2.82)
	Cognitive disorder	34	2.04 (17.97)	2.04 (1.46–2.86)
Respiratory, thoracic and mediastinal disorders	Pneumonitis	67	7.51 (376.94)	7.53 (5.92–9.57)
	Interstitial lung disease	72	4.34 (185.18)	4.36 (3.46–5.49)
	Pulmonary embolism	82	2.28 (58.99)	2.29 (1.84–2.84)
General disorders and administration site conditions	Mucosal inflammation	53	5.78 (208.93)	5.79 (4.42–7.58)
	Malaise	327	2.02 (168.49)	2.03 (1.82–2.27)
Blood and lymphatic system disorders	Leukopenia	67	3.79 (137.80)	3.80 (2.99–4.84)
	Neutropenia	97	2.09 (55.20)	2.09 (1.72–2.56)
Skin and subcutaneous Tissue disorders	Alopecia	226	3.13 (328.54)	3.15 (2.77–3.60)
	Night sweats	35	3.19 (52.47)	3.19 (2.29–4.45)
Psychiatric disorders	Insomnia	215	2.20 (141.12)	2.21 (1.93–2.53)
	Sleep disorder	49	2.07 (27.06)	2.07 (1.56–2.74)
Hepatobiliary disorders	Hepatic function abnormal	43	3.38 (71.97)	3.38 (2.51–4.57)
	Hepatic failure	31	2.78 (35.26)	2.78 (1.95–3.96)

Ear and labyrinth disorders	Hypoacusis	60	3.48 (106.03)	3.49 (2.71–4.49)
	Vertigo	47	2.10 (27.08)	2.10 (1.58–2.80)
Gastrointestinal disorders	Stomatitis	103	4.89 (318.38)	4.91 (4.04–5.96)
Vascular disorders	Hot flush	261	10.46 (2226.43)	10.58 (9.36–11.95)
Metabolism and nutrition disorders	Diabetes mellitus	77	2.69 (81.70)	2.69 (2.15–3.37)
Eye disorders	Cataract	53	2.62 (53.17)	2.63 (2.01–3.44)
Reproductive system and breast disorders	Vaginal haemorrhage	48	2.92 (60.68)	2.93 (2.20–3.89)

Abbreviations: AE: adverse event; SOC: system organ class; ROR: reporting odds ratio; CI: confidence interval; RRR: proportional reporting ratio.

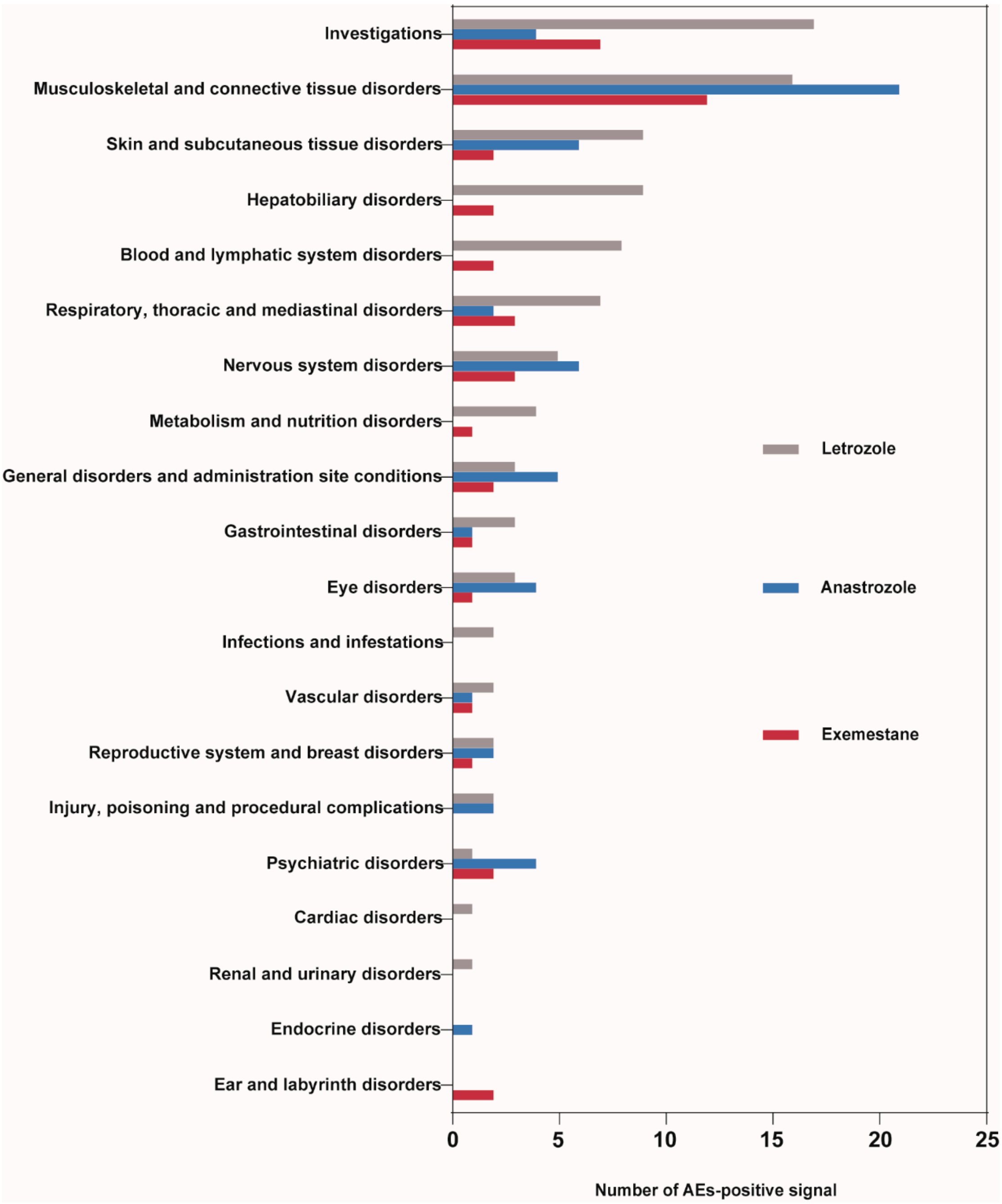

Figure S1. The number of involving system signals for three AIs. Abbreviations: AI: aromatase inhibitor.

Subgroup	AE	n	ROR(95% CI)
Medical professionals	Skin hypopigmentation	51	52.42(39.3-69.93)
	Uterine polyp	36	28.46(20.35-39.82)
	Trigger finger	39	19.61(14.24-27)
	Bone lesion	51	14.16(10.72-18.7)
	Onychoclasis	42	11.68(8.6-15.86)
	Neutropenia	1470	8.72(8.28-9.19)
	Hot flush	296	8.63(7.69-9.69)
	Leukopenia	527	8.24(7.56-8.99)
	Polyneuropathy	119	8.72(7.27-10.45)
	Bone pain	295	7.65(6.82-8.59)
Non-medical professionals	Neutropenia	366	34.93(31.45-38.79)
	Trigger finger	67	36.62(28.69-46.74)
	Leukopenia	106	22.14(18.25-26.84)
	Vulvovaginal dryness	32	19.82(13.97-28.12)
	Neutrophil count decreased	67	13.03(10.24-16.59)
	Gamma-glutamyltransferase increased	32	11.59(8.18-16.43)
	Bone pain	213	8.68(7.58-9.93)
	Hot flush	285	8.21(7.3-9.24)
	Febrile neutropenia	36	9.84(7.09-13.67)
	White blood cell count decreased	218	7.27(6.36-8.31)

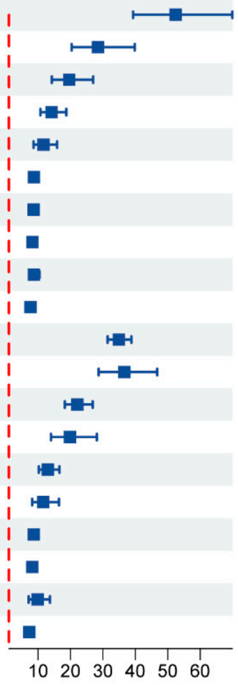

Figure S2. Subgroup analysis of letrozole use showing the forest plot of ROR (95% CI) for the top 10 AEs with the strongest signal intensity. Abbreviations: AE: adverse event; ROR: reporting odds ratio; CI: confidence interval.

Subgroup	AE	n	ROR(95% CI)
Medical professionals	Neutropenia	1470	8.72(8.28-9.19)
	Arthralgia	721	2.56(2.38-2.75)
	Anaemia	547	2.64(2.42-2.87)
	Leukopenia	527	8.24(7.56-8.99)
	Thrombocytopenia	452	3.14(2.86-3.45)
	Alopecia	359	3.54(3.19-3.93)
	Hot flush	296	8.63(7.69-9.69)
	Bone pain	295	7.65(6.82-8.59)
	Electrocardiogram qt prolonged	224	4.6(4.04-5.25)
	Alanine aminotransferase increased	213	2.69(2.35-3.08)
Non-medical professionals	Fatigue	689	2.07(1.92-2.24)
	Arthralgia	559	3.45(3.17-3.75)
	Neutropenia	366	34.93(31.45-38.79)
	Alopecia	339	3.29(2.95-3.66)
	Hot flush	285	8.21(7.3-9.24)
	Myalgia	222	3.46(3.03-3.95)
	White blood cell count decreased	218	7.27(6.36-8.31)
	Bone pain	213	8.68(7.58-9.93)
	Anaemia	115	2.64(2.2-3.17)
	Leukopenia	106	22.14(18.25-26.84)

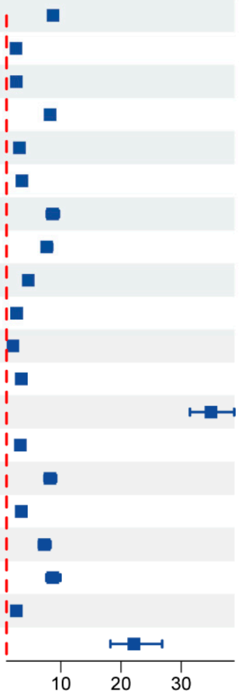

Figure S3. Subgroup analysis of letrozole use showing the forest plot of ROR (95% CI) for the top 10 AEs with the highest frequency. Abbreviations: AE: adverse event; ROR: reporting odds ratio; CI: confidence interval.

Subgroup	AE	n	ROR(95% CI)
Medical professionals	Carpal tunnel syndrome	37	29.01(20.97-40.13)
	Hot flush	97	18.65(15.26-22.8)
	Joint stiffness	31	11.16(7.84-15.89)
	Bone pain	57	9.69(7.46-12.58)
	Arthralgia	246	5.87(5.17-6.67)
	Arthritis	41	5.65(4.16-7.69)
	Interstitial lung disease	43	4.7(3.48-6.35)
	Myalgia	68	3.63(2.86-4.61)
	Deep vein thrombosis	31	2.95(2.07-4.2)
	Muscular weakness	34	2.88(2.06-4.04)
Non-medical professionals	Trigger finger	54	47.86(36.5-62.75)
	Vulvovaginal dryness	47	48.01(35.91-64.18)
	Hot flush	388	18.58(16.78-20.56)
	Carpal tunnel syndrome	50	12.9(9.76-17.04)
	Bone pain	170	11.31(9.72-13.16)
	Osteopenia	61	12.01(9.33-15.46)
	Arthritis	202	9.42(8.2-10.83)
	Osteoporosis	106	8.44(6.97-10.22)
	Body height decreased	36	9.58(6.9-13.29)
	Mood altered	54	7.34(5.62-9.59)

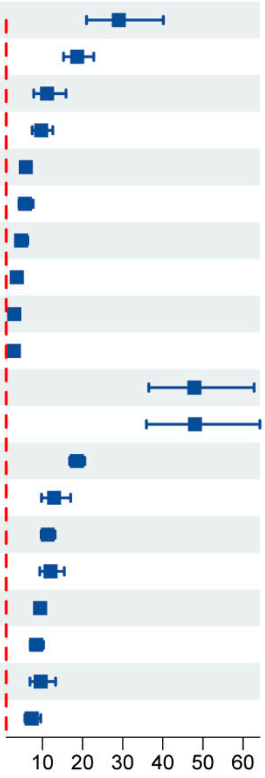

Figure S4. Subgroup analysis of anastrozole use showing the forest plot of ROR (95% CI) for the top 10 AEs with the strongest signal intensity. Abbreviations: AE: adverse event; ROR: reporting odds ratio; CI: confidence interval.

Subgroup	AE	n	ROR(95% CI)
Medical professionals	Arthralgia	246	5.87(5.17-6.67)
	Hot flush	97	18.65(15.26-22.8)
	Myalgia	68	3.63(2.86-4.61)
	Pain in extremity	64	2.24(1.75-2.86)
	Bone pain	57	9.69(7.46-12.58)
	Depression	46	2.25(1.68-3)
	Interstitial lung disease	43	4.7(3.48-6.35)
	Weight increased	42	2.14(1.58-2.89)
	Arthritis	41	5.65(4.16-7.69)
	Alopecia	39	2.53(1.84-3.46)
Non-medical professionals	Arthralgia	593	6.08(5.6-6.6)
	Hot flush	388	18.58(16.78-20.56)
	Pain in extremity	236	2.86(2.51-3.25)
	Insomnia	217	2.66(2.32-3.04)
	Arthritis	202	9.42(8.2-10.83)
	Weight increased	198	3.21(2.79-3.69)
	Alopecia	182	2.87(2.48-3.32)
	Depression	175	2.62(2.26-3.05)
	Bone pain	170	11.31(9.72-13.16)
	Myalgia	140	3.55(3.01-4.2)

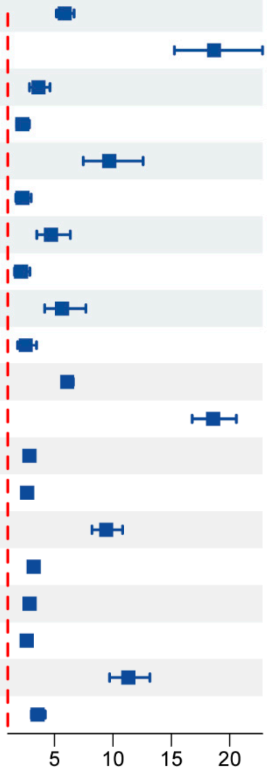

Figure S5. Subgroup analysis of anastrozole use showing the forest plot of ROR (95% CI) for the top 10 AEs with the highest frequency. Abbreviations: AE: adverse event; ROR: reporting odds ratio; CI: confidence interval.

Subgroup	AE	n	ROR(95% CI)
Medical professionals	Hot flush	89	10.56(8.56-13.01)
	Pneumonitis	55	7.51(5.76-9.79)
	Bone pain	68	7.17(5.64-9.1)
	Stomatitis	80	6.14(4.93-7.65)
	Mucosal inflammation	52	6.36(4.84-8.36)
	Vaginal haemorrhage	30	5.72(3.99-8.18)
	Interstitial lung disease	70	4.76(3.76-6.02)
	Gamma-glutamyltransferase increased	31	4.29(3.02-6.11)
	Arthralgia	228	3.33(2.92-3.8)
	Leukopenia	58	3.65(2.82-4.73)
Non-medical professionals	Hot flush	164	11.21(9.61-13.09)
	Bone pain	113	10.89(9.04-13.12)
	Arthralgia	351	5.19(4.66-5.77)
	Hypoacusis	49	4.27(3.22-5.65)
	Arthropathy	40	4.29(3.14-5.86)
	Myalgia	103	3.8(3.13-4.61)
	Arthritis	59	3.95(3.06-5.1)
	Alopecia	154	3.54(3.02-4.15)
	Osteoporosis	32	3.67(2.6-5.2)
	Malaise	229	2.65(2.32-3.02)

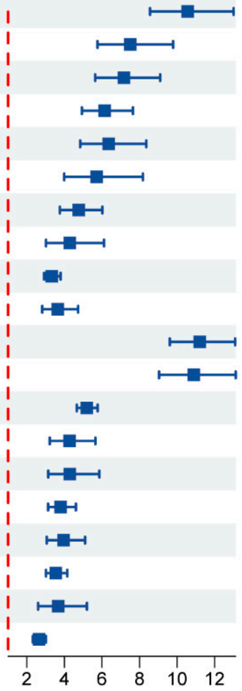

Figure S6. Subgroup analysis of exemestane use showing the forest plot of ROR (95% CI) for the top 10 AEs with the strongest signal intensity. Abbreviations: AE: adverse event; ROR: reporting odds ratio; CI: confidence interval.

Subgroup	AE	n	ROR(95% CI)
Medical professionals	Arthralgia	228	3.33(2.92-3.8)
	Hot flush	89	10.56(8.56-13.01)
	Stomatitis	80	6.14(4.93-7.65)
	Insomnia	72	2.15(1.71-2.71)
	Interstitial lung disease	70	4.76(3.76-6.02)
	Alopecia	70	2.82(2.23-3.57)
	Myalgia	70	2.31(1.83-2.93)
	Bone pain	68	7.17(5.64-9.1)
	Pulmonary embolism	62	2.59(2.02-3.32)
	Cerebrovascular accident	59	2.15(1.66-2.78)
Non-medical professionals	arthralgia	351	5.19(4.66-5.77)
	malaise	229	2.65(2.32-3.02)
	hot flush	164	11.21(9.61-13.09)
	alopecia	154	3.54(3.02-4.15)
	insomnia	135	2.4(2.02-2.84)
	pain in extremity	132	2.31(1.95-2.75)
	bone pain	113	10.89(9.04-13.12)
	myalgia	103	3.8(3.13-4.61)
	memory impairment	67	2.14(1.68-2.72)
	arthritis	59	3.95(3.06-5.1)

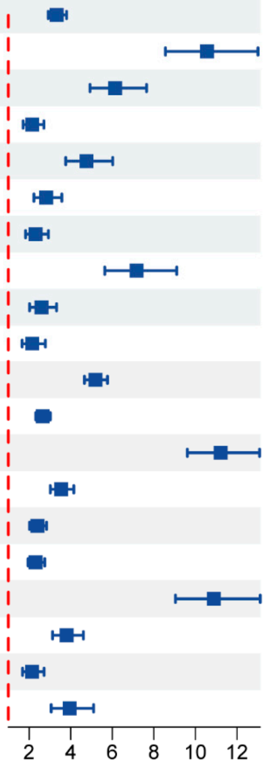

Figure S7. Subgroup analysis of exemestane use showing the forest plot of ROR (95% CI) for the top 10 AEs with the highest frequency. Abbreviations: AE: adverse event; ROR: reporting odds ratio; CI: confidence interval.
